# Risk factors for Hirschsprung-associated enterocolitis following Soave: a retrospective study over a decade

**DOI:** 10.1186/s12887-022-03692-6

**Published:** 2022-11-10

**Authors:** Chuanping Xie, Jiayu Yan, Zhiyi Zhang, Wang Kai, Zengmeng Wang, Yajun Chen

**Affiliations:** 1 Department of General Surgery, Beijing Children’s Hospital, Capital Medical University, National Center for Children’s Health, Beijing, China; 2 Present address: Beijing, China

**Keywords:** Hirschsprung disease, Enterocolitis, Risk factors

## Abstract

**Background:**

Hirschsprung-associated enterocolitis (HAEC), one of the most significant causes of morbidity and mortality for patients with Hirschsprung disease (HSCR), can occur before and after radical surgery. This study aims to identify the risk factors for HAEC before and after Soave.

**Methods:**

A retrospective study of 145 patients with HSCR treated by transanal or combination with laparoscopic or laparotomy Soave procedure between January 2011 and June 2021 was performed. Data were retrieved from the medical records. HAEC was defined as the presence of clinical signs of bowel inflammation and requiring treatment with intravenous antibiotics and rectal irrigation for at least two days in the outpatient or inpatient department. Univariate analysis and multivariate regression models were used to identify risk factors for developing pre-and postoperative HAEC.

**Results:**

The incidence of pre-and postoperative HAEC was 24.1% and 20.7%, respectively. More than 90% of the patients with the first episode of postoperative HAEC occurred within the first year after Soave. Long-segment aganglionosis was the independent risk factor for developing preoperative HAEC ([OR] 5.8, Cl 2.4–14.2, *p* < 0.001), while the history of preoperative HAEC was significantly associated with developing postoperative HAEC ([OR] 4.2, Cl 1.6–10.8, *p* = 0.003).

**Conclusions:**

Long-segment aganglionosis was the independent risk factor for the development of preoperative HAEC, and the history of preoperative HAEC was strongly associated with developing HAEC after Soave.

**Level of Evidence:**

Level III

## Introduction

Hirschsprung disease (HSCR), known as aganglionosis, is a common cause of intestinal obstruction in children. It approximately occurs in one of 5000 newborns with an overall male: female ratio of 4:1 [1]. Despite the advancement of surgical management, patients remain at risk of developing a life-threatening condition, presenting as abdominal distension, fever, diarrhea, obtunded, and ultimate sepsis [2, 3]. Bill and Chapman defined these symptoms as Hirschsprung-associated enterocolitis (HAEC) [3].

HAEC, the most significant cause of morbidity and mortality for patients with HSCR, can occur before and after the radical operation of HSCR [4, 5]. Based on recent studies, several hypotheses were involved in the pathogenesis of HAEC, including impaired mucosal immunity, immature barrier defence, dysbiosis of the intestinal microbiota, and translocation of bacteria [2, 6]. Pastor et al. primarily attempted to standardize the definition of HAEC and developed a score for HAEC with a cutoff of 10 [3]. However, it was believed that Pastor’s scoring system failed to diagnose milder HAEC [7].

Some studies have reported risk factors for preoperative and postoperative HAEC, including body weight, age of diagnosis, female sex, congenital malformation, long-segment aganglionosis, and surgical methods (Soave or Duhamel) [8–11]. However, the results of these studies were partially controversial. Besides, in previous studies, patients with HSCR received different surgical methods, leading to certain deviations in developing HAEC [10, 12–14]. The purpose of the current study was to describe the clinical characteristics of the patients with HAEC and evaluate the risk factors for developing HAEC before and after Soave.

## Materials and methods

### Patient selection

Approved by the Ethics Committee of Beijing Children’s Hospital, we reviewed the medical records of consecutive patients with HSCR who underwent radical surgery at Beijing Children’s Hospital, National Center for Children’s Health, between January 2011 and June 2021. Patients who underwent radical surgery in other hospitals or patients without histopathological confirmation of HSCR were excluded. All patients underwent Soave pull-through procedure by the same surgeon team. Medical records of all patients were analyzed, including patient characteristics (gender, birth weight, gestational age, congenital malformation, age at diagnosis), surgical details, rates of HAEC, and postoperative complications (anastomotic leak). HSCR was divided into two types based on the length of the aganglionic segment: the short-segment type was defined as aganglionosis extending to the rectosigmoid, and the long-segment type as aganglionosis extending proximal to the sigmoid. The Soave pull-through procedures performed in our study were divided into two types: the operation performed by transanal endorectal pull-through (TERPT) and in combination with laparoscopic or laparotomy-assisted colonic mobilization (LERPT).

Unfortunately, it was impossible to collect all the data necessary for diagnosing HAEC by Pastor’s scoring system due to the retrospective study in nature. Therefore, the following definition of HAEC was based on clinical symptoms and treatment strategies [13], including: (a) presence of clinical signs of bowel inflammation, such as abdominal distension, diarrhea with explosive stool, fever, lethargy, a dilated loop of bowel and even sepsis, (b) that required with intravenous antibiotics and rectal irrigations at least two days in the outpatient or inpatient department.

A comparative study was performed to analyze risk factors between patients with pre-and postoperative HAEC and those without HAEC. The risk factors for preoperative HAEC were analyzed by gender, birth weight, gestational age, congenital malformation, age at diagnosis, age at surgery, weight at surgery, and length of aganglionosis. The risk factors for postoperative HAEC were analyzed by gender, birth weight, gestational age, congenital malformation, age at diagnosis, age at surgery, weight at surgery, length of aganglionosis, the presence of preoperative HAEC, surgical approach, and the presence of temporary ostomy.

### Statistical analysis.

Statistical analysis was conducted using IBM SPSS Statistics for Statistics ver. 26.0 Software. Data were presented as frequency or median (interquartile range, IQR). All the statistical tests were two-sided, with a significant level of *p* < 0.05. On univariate analysis, Chi-squared tests or Fisher’s exact tests were applied for univariable analysis for qualitative variables and the independent sample t-test or Wilcoxon rank-sum test (Mann–Whitney) for continuous variables. Multivariable logistic regression analysis included significant variables (*p* < 0.05) from univariate analysis.

## Results

### Patient demography

**Fig. 1:**
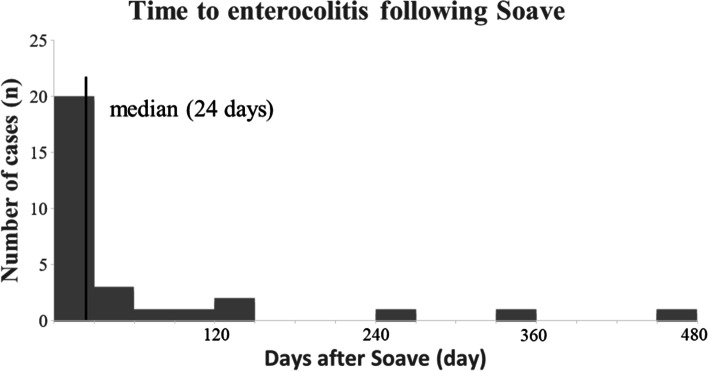
Days from Soave to the first episode of postoperative HAEC. The median is shown with a solid back line

From January 2011 to June 2021, 145 patients were included in our study, including 121 males and 24 females. Of these patients, 35 (35/145, 24.1%) had preoperative HAEC, and 30 (30/145, 20.7%) had postoperative HAEC. The baseline characteristics of all 145 patients are presented in Table 1. During the follow-up, more than 90% of the patients with the first episode of postoperative HAEC occurred within a year following pull-through with a median time of 24 days (Fig. 1).

**Table 1:** Characteristics of patients with Hirschsprung disease

Variable	*N* = 145
Sex	
Male	121 (83.4)
Female	24(16.6)
Gestational age	
Preterm (< 37 weeks)	5 (3.4)
Term (≥ 37 weeks)	140 (96.6)
Birthweight in kilograms, mean (SD)	3.31 ± 0.47
Low birthweight (< 2500 g)	6 (4.1)
Normal birthweight (≥ 2500 g)	139 (95.9)
Congenital malformation	11 (7.6)
Age at diagnosis in months	13.5 [6.8, 44.0]
0–1 year of age	66 (45.5)
> 1 year of age	79 (54.5)
Length of aganglionosis^a^	
Short	116 (80.0)
Long	29 (20.0)
Age at surgery in month	20.0 [10.0, 47.5]
0–1 year of age	46 (31.7)
> 1 year of age	99 (68.3)
Weight at first surgery in kilogram	11.7 [9.0, 15.9]
Approach of operation technique	
Transanal only	92 (63.4)
Laparotomy + transanal	43 (29.7)
Laparoscopic + transanal	10 (6.9)
Temporary ostomy before radical surgery	9 (6.2)
HAEC	
Preoperative	35 (24.1)
Postoperative	30 (20.7)
Recurrent postoperative	11 (7.6)
Complication	
Anastomosis leak	8 (5.5)

Data are presented as median [IQR, interquartile range] and frequency (%)

Length of aganglionosis^a^: Long = longer than rectosigmoid, total colonic forms included (total colonic forms *n* = 9)

### Risk factors for preoperative HAEC

Univariate analysis was performed to compare patients with preoperative HAEC (*n* = 35) to patients without preoperative HAEC (*n* = 110) (Table 2). HSCR patients with preoperative HAEC were diagnosed at a younger age (*p* = 0.027). Compared with short-segment aganglionosis, long-segment aganglionosis was a vital risk factor for developing preoperative HAEC ([OR] 6.3, Cl 2.6–15.2, *p* < 0.001). However, there was no significant association between the patients with preoperative HAEC and those without preoperative HAEC regarding sex, gestational age, weight at birth, age at radical surgery, and weight at radical surgery. On multivariable logistic regression analysis, only the length of aganglionosis was significantly associated with preoperative HAEC ([OR] 5.8, Cl 2.4–14.2, *p* < 0.001) adjusted by the age of diagnosing HSCR, (Table 3).

**Table 2:** Comparison of HSCR patients with or without preoperative HAEC

Factors	Preoperative HAEC (*n* = 35)	Without preoperative HAEC (*n* = 110)	*P*-value	OR
Male sex			0.440	1.7 [0.5, 5.4]
Male	31 (88.6)	90 (81.8)		
Female	4 (11.4)	20 (18.2)		
Gestational age				
Preterm (< 37 weeks)	2 (5.7)	3 (2.7)	0.594	2.1 [0.3, 13.5]
Term (≥ 37 weeks)	33 (94.3)	107 (97.3)		
Age at diagnosis (months)	10.5 [0, 24.8]	18.0 [7.0, 47.8]	0.027	
Age at diagnosis				
0–1 year of age	21 (60.0)	45 (40.9)	0.054	2.2 [1.0, 4.7]
> 1 year of age	14 (40.0)	65 (59.1)		
Age at radical surgery (months)	18.5 [10.0, 37.3]	23.5 [10.0, 50.8]	0.329	
Age at radical surgery				
0–1 year of age	13 (37.1)	33 (30.0)	0.532	1.4 [0.6, 3.1]
> 1 year of age	22 (62.9)	77 (70.0)		
Weight at birth (kg)	3.42 ± 0.45	3.27 ± 0.47	0.537	
Weight at radical surgery (kg)	10 [8.0, 10.3]	12 [9.0, 16.0]	0.093	
Congenital malformations				
Yes	4 (11.4)	7 (6.4)	0.462	1.9 [0.5, 6.9]
No	31 (88.6)	103 (93.6)		
Length of aganglionosis^a^				
Short	19 (54.3)	97 (88.2)	< 0.001	6.3 [2.6, 15.2]
Long	16 (45.7)	13 (11.8)		

Data are presented as frequency (%) for qualitative variables and median [IQR] for continuous variables

Length of aganglionosis^a^: Long = longer than rectosigmoid, total colonic forms included (total colonic forms *n* = 9)

**Table 3:** Factors predicting preoperative HAEC in multivariate model

Risk factors	Odds ratio	Confidence interval	*p*-value
Age at diagnosis	1.0	1.0–1.02	0.295
length of aganglionosis	5.8	2.4–14.2	< 0.001

### Risk factors for postoperative HAEC

Table 4 shows the risk factors between patients with postoperative HAEC (*n* = 30) and patients without postoperative HAEC (*n* = 115). Based on univariate analysis, the length of aganglionosis ([OR] 3.1, Cl 1.3–7.6, *p* = 0.019), the history of preoperative HAEC ([OR] 4.8, Cl 2.0–11.3, *p* < 0.001) and surgical approach ([OR] 2.8, Cl 1.2–6.3, *p* = 0.013) were significantly associated with developing postoperative HAEC. No significant association was found between patients who had postoperative HAEC and those who did not, in terms of sex, gestational age, congenital malformation, birth weight, age at diagnosis, age at radical surgery, weight at radical surgery, and presence of a temporary ostomy. However, only the history of preoperative HAEC was the independent factor for developing postoperative HAEC adjusted by the length of aganglionosis and surgical approach on multivariable logistic regression analysis (Table 5).

**Table 4:** Comparison of HSCR patients with or without postoperative HAEC

Factors	Postoperative HAEC (*n* = 30)	Without postoperative HAEC (*n* = 115)	*P*-value	OR [95% Cl]
Male sex				
Male	24 (80.0)	97 (84.3)	0.585	0.7 [0.3, 2.1]
Female	6 (20.0)	18 (15.7)		
Gestational age				
Preterm (< 37 weeks)	1 (3.3)	4 (3.5)	> 0.999	1.0 [0.1, 8.9]
Term (≥ 37 weeks)	29 (96.7)	111 (96.5)		
Age at diagnosis (months)	12 [1.0, 38.0]	15 [7.0, 45.5]	0.503	
Age at radical surgery (months)	25 [8.3, 38.8]	19 [10.0, 48.0]	0.736	
Weight at birth (kg)	3.4 [3.0, 3.6]	3.3 [3.0, 3.6]	0.392	
Weight at radical surgery (kg)	12.5 [8.3, 38.8]	19 [10.0, 48.0]	0.842	
Congenital malformation				
Yes	3 (10.0)	8 (7.0)	0.698	1.5 [0.4, 6.0]
No	27 (90.0)	107 (93.0)		
Length of aganglionosis^a^				
Short	19 (63.3)	97 (84.3)	0.019	3.1 [1.3, 7.6]
Long	11 (36.7)	18 (15.7)		
Preoperative HAEC				
Yes	15 (50.0)	20 (17.4)	< 0.001	4.8 [2.0, 11.3]
No	15 (50.0)	95 (82.6)		
Surgical approach				
TERPT	13 (43.3)	79 (68.7)	0.013	2.8 [1.2, 6.3]
LERPT	17 (56.7)	37 (32.2)		
Temporary ostomy				
Yes	3 (10.0)	6 (5.2)	0.393	2.0 [0.5, 8.6]
No	27 (90.0)	109 (94.8)		

Data are presented as frequency (%) for qualitative variables and median [IQR] for continuous variables

Length of aganglionosis^a^: Long = longer than rectosigmoid, total colonic forms included (total colonic forms *n* = 9)

**Table 5:** Risk factors for developing recurrent postoperative HAEC

Risk factors	Odds ratio	Confidence interval	*P*-value
Length of aganglionosis	1.1	0.3–4.0	0.920
Surgical approaches	2.3	0.8–6.5	0.132
History of preoperative HAEC	4.2	1.6–10.8	0.003

## Discussion

There have been several studies on the risk factors of pre-and postoperative HAEC [8, 10, 12–15]. However, few studies have systematically analyzed the risk factors for HAEC before and after Soave, which might be related to fewer cases in other centers. Therefore, we performed one of the largest single-center retrospective studies to identify the risk factors for HAEC before and after Soave. HAEC can rapidly progress to septicemia, resulting in mortality in patients with HSCR [8, 16]. The incidence of pre-and postoperative HAEC ranged widely, from 6 to 50% and 2% to 35%, which might be related to heterogeneity in case definitions and differences in clinical characteristics of the cohorts [13, 17]. In our center, the age for radical surgery was significantly older than in most previous reports [8, 12]. Nearly 70% of HSCR patients underwent radical surgery after 1 year because they usually suffered from less severe symptoms, which could be improved through conservative treatments. Most of them would not be transferred to hospitals for radical surgery until presenting more severe symptoms. It could also explain why the incidence of preoperative HAEC (24.1%) was relatively higher than most previous reports [8, 12, 15].

Our study’s univariate analysis revealed that the earlier age at diagnosing HSCR and long-segment aganglionosis segment were associated with developing preoperative HAEC. However, regression analysis identified that only the long-segment aganglionosis segment was the independent risk factor for the development of preoperative HAEC. It could be explained that patients with long-segment aganglionosis tended to have more severe symptoms at a younger age, leading to an earlier age of diagnosing HSCR [18, 19]. The mechanism of an increased risk factor for developing HAEC was as follows: first, the longer aganglionosis tends to produce the proximal bowel obstruction and generate more significant intraluminal pressure, leading to a higher vulnerability to bacterial stasis and intestinal dysmotility; second, it also involves the impairment of the bowel immune system, resulting in pathogenic bacteria overgrowth and possible bacteria translocation [8, 10]. Previous studies suggested that congenital malformations and lower birth weight were associated with preoperative HAEC [8, 15]. In contrast to these studies, our study did not support these results, which might be related to the absence of severe congenital malformations (such as congenital cardiac or neurologic anomalies and chromosome abnormalities) and the low frequency of premature infants.

In our study, the incidence of postoperative HAEC was 20.7%, and more than 90% of the patients with the first episode of postoperative HAEC occurred within a year following the pull-through [12, 20]. Univariate analysis revealed that the length of aganglionosis, surgical approaches, and the history of preoperative enterocolitis were significantly associated with developing postoperative HAEC. However, multivariable logistic regression analysis established that only the history of preoperative HAEC was strongly associated with the increased risk of postoperative HAEC since patients with long-segment aganglionosis tended to present more severe symptoms before radical surgery [2, 10, 20]. A hypothesis proposed that patients with preoperative HAEC episodes could change resultant short-chain fatty, alter the composition of bacteria, and reduce the diversity of fungi, leading to dysbiosis in the gut microbial ecosystem, even after radical surgery [21–23]. The changes in intestinal microbiota made patients susceptible to the development of further episodes of HAEC [10, 22]. Previous studies also suggested that a higher pathological HAEC score of the resected colon in HSCR patients could increase the risk of developing further episodes of enterocolitis, and the severity of enterocolitis in the transitional zone was the most significant factor [24, 25]. To reduce the incidence of postoperative HAEC, it was recommended that radical surgery be performed at an early age before the presence of severe symptoms [13]. Earlier radical surgery for HSCR patients could improve intestinal microbiota and reduce the incidence of preoperative enterocolitis [13, 26]. For patients with preoperative HAEC, conservation treatment should be given first, such as antibiotics and rectal irrigations. However, a temporary enterostomy might be needed for the patients who failed to improve with non-operative management, and radical surgery should be performed until the patients’ conditions are stable [2]. Some previous studies proposed that long-segment aganglionosis could increase the risk of developing HAEC after radical surgery [10, 19], but our study did not demonstrate it on multivariable logistic regression. The possible reasons might be as follow. On the one hand, we were prone to remove all the lesion bowel (including spasm segment, transitional and proximal dilated zones) to make the proximal pull-through bowel normal in terms of pathology and morphology, effectively reducing residual neuronal incidence dysplasia in the proximal bowel [27]. On the other hand, our patients began routine anal dilatation two weeks after radical surgery, which could reduce the frequency of anastomotic stricture and assist patients in developing regular bowel movements by stimulating the anus [16, 28, 29].

In our center, there were 8 patients with recurrent episodes of HAEC after Soave. Four patients had an uneventful recovery after the treatment of bowel rest, rectal irrigations, and broad-spectrum antibiotics, and four patients underwent a repeated pull-through due to residual intestinal neuronal dysplasia. It is critical to evaluate for an anatomic/pathological or functional obstruction and carefully examine patients with recurrent HAEC after Soave, such as barium enema, anorectal pressure measurement, or rectal biopsy [2, 20]. Although we tend to remove all the lesion bowel, some patients suffered recurrent enterocolitis after Soave due to residual intestinal neuronal dysplasia, and repeated pull-through was required. Moreover, for patients with obstructive symptoms due to non-relaxation of the internal anal sphincter, it has been proposed that intrasphincteric botulinum toxin injection could alleviate obstructive defecation problems following pull-through with mild adverse effects [2, 30, 31].

The study has some limitations. One of the limitations of our study is the lack of a standardized definition for diagnosing HAEC. Although Pastor et al. attempted to standardize the definition of HAEC and developed a scoring system based on Delphic analysis, collecting the data necessary for Pastor’s scoring system was challenging because part of our cohort had been treated with antibiotics or rectal irrigation before validating the scores. Besides, our case definition might be strict for diagnosing HAEC. Some patients with milder HAEC treated with oral antibiotics at home without hospitalization might be excluded from our study, leading to a potential selection bias. Last but not least, Our study is retrospective and single-center, which could also result in a particular deviation.

## Conclusions

In conclusion, long-segment aganglionosis was the independent risk factor for developing preoperative HAEC, while the history of preoperative HAEC was strongly associated with developing HAEC after Soave.

## Data Availability

All data generated or analyzed during this study are included in this manuscript.

## Abbreviations

HAEC: Hirschsprung-associated enterocolitis
HSCR: Hirschsprung disease
